# The need for standardized tracking of investments in digital health

**DOI:** 10.2471/BLT.25.293752

**Published:** 2026-05-11

**Authors:** Kirsten M Mathieson, Mathilde Forslund

**Affiliations:** a Transform Health, c/o Medicus Mundi Switzerland, Marbruchstrasse 34, CH-4056 Basel, Switzerland.

Digital health transformation plays a vital role in enabling progress towards universal health coverage (UHC). This transformation encompasses the use of digital technologies to strengthen health systems, improve service delivery and enhance health outcomes. Tools include electronic health records, telemedicine, health information exchanges, mobile applications and data analytics, alongside the supporting infrastructure, governance frameworks and policies.

Although the global community recognizes the potential of digital health, the funding landscape for digital transformation is weakened by challenges, including insufficient, fragmented and poorly aligned or targeted investment, together with a lack of information on current funding.^1^ Investments originate from multiple sources, including government budgets, for example for health and information and communications technology, development partners, philanthropic organizations and the private sector. This complexity, combined with inconsistent definitions of investment areas, makes it difficult to determine how much is being invested, where funding is concentrated and whether critical gaps remain.

The absence of standardized categories and reporting mechanisms as well as fragmented data limit transparency and accountability. Policy-makers often lack reliable information to assess needs, coordinate financing and evaluate effectiveness, or to make the case for additional financial resources. Without consistent tracking, digital health spending may remain invisible within broader health budgets or vertical programmes, reducing opportunities for strategic planning and system-wide coordination.

The importance of transparency, accountability and reporting and coordination of investment is widely recognized in global policy discussions, including the Busan Partnership, Addis Ababa Action Agenda and Lusaka Agenda. The International Monetary Fund also emphasizes reliable and transparent national budgeting and reporting for improved accountability and public trust.^2^

In this article, we argue for improved tracking of digital health investments to strengthen coordination, transparency and accountability. We examine current gaps in tracking digital health financing, draw lessons from other areas of global health investment, and propose the development of a global digital health investment taxonomy to enable standardized classification and reporting across stakeholders. Our proposal takes forward recommendations from the 2024 Group of 20 Health Ministers Declaration, which called on countries and development partners to support the tracking of digital health investments.

## Investment tracking challenges

In 2022, Transform Health published a conceptual framework to guide investments needed for the equitable, inclusive and sustainable digital transformation of health systems in low- and middle-income countries.^1^ The report highlighted a notable challenge in the digital health funding landscape, the lack of comprehensive information on financial resources allocated to digital health. Digital health spending is frequently embedded within broader health programmes, disease-specific or other vertical health programme areas, or information and communications technology investments, making it difficult to isolate and analyse. As a result, policy-makers lack a comprehensive picture of funding flows.

A major challenge is the absence of a globally agreed taxonomy for digital health investments. Moreover, existing reporting systems, such as the Organisation for Economic Co-operation and Development’s (OECD) Development Assistance Committee Creditor Reporting System and System of Health Accounts do not capture the specificity needed for digital health tracking. A 2024 consultation found that only five out of 32 organizations surveyed (covering civil society, implementing and/or technical partners, intergovernmental organizations, multilateral donor agencies, academia and private foundations) have systems to track digital health funding,^3^ while many governments and donors do not report such spending through existing mechanisms, with data fragmented across donor databases.

The digital health landscape is similarly fragmented, an issue highlighted in the Global Strategy on Digital Health 2020–2025.^4^ For example, a study revealed 738 distinct digital health interventions in Africa over a decade.^5^ In many countries, a large proportion of digital health investment is driven by external partners rather than national priorities, which can create misalignment with country strategies and needs.^6^ Without better visibility of investments, such fragmentation and misalignment leads to duplication, inefficiencies and missed opportunities to support a more sustainable digital health transformation.

## The case for improved tracking

The global community increasingly recognizes the value of digital health in advancing UHC, and the importance of transparency in digital health investment to improve coordination, alignment and prioritization. We suggest five arguments for the improved tracking of financing for digital health. First, tracking investments would improve financing effectiveness and enable policy-makers to assess how investments contribute to health system strengthening and UHC. Second, better financial data would allow governments and development partners to analyse spending patterns, identify underfunded areas and allocate resources more strategically, supporting evidence-based decision-making. Third, comprehensive tracking would reveal financing gaps and help ensure that investments align with national health priorities and address critical health needs. Fourth, better transparency would strengthen coordination among stakeholders and reduce fragmentation and duplication while enabling more strategic allocation of resources and complementary investments. Finally, more transparent reporting would allow governments, oversight bodies and citizens to assess how resources are used, thereby enhancing accountability and public trust. 

## Lessons from other health areas

The value of standardized classification and transparent reporting is well established in other areas of global health financing. Studies analysing development assistance for health show that improved tracking enables donors and policy-makers to identify funding trends and respond to gaps.^7^^,^^8^

Researchers and international agencies have developed dedicated reporting codes or tracking methods for several global health areas, including for human immunodeficiency virus (HIV)/acquired immunodeficiency syndrome (AIDS); malaria; reproductive, maternal, newborn and child health; nutrition; and noncommunicable diseases. These efforts have improved visibility of funding flows and investment gaps and contributed to sustained political attention and commitments. For example, the OECD Development Assistance Committee’s code for noncommunicable diseases enables granular tracking of donor funding, revealing investment gaps, supporting evidence-based allocation and increasing political visibility. Similarly, tracking of donor spending for HIV/AIDS has provided evidence on funding trends, burden-sharing among donors and progress towards global targets, generating sustained political attention and financing commitments.^9^ The Muskoka2 method and the Global Financing Facility’s resource mapping and expenditure tracking approach further show how cross-cutting health investments can be captured within existing frameworks, which has improved visibility of official development assistance and domestic financing, strengthened coordination and mobilized additional resources.^10^^–^^12^

These examples offer several lessons for digital health. For example, investment tracking improves transparency and accountability, allowing stakeholders to identify funding gaps, monitor alignment with priorities and assess progress towards goals. Moreover, standardized definitions and dedicated tracking codes enable comparability, providing greater visibility within broader budgets and funding. Finally, systematic tracking can raise political awareness.

While digital health shares many similarities with these investment areas, it presents additional challenges that makes dedicated tracking especially important. Digital health investments span multiple programmes, ministries and funding streams, and they evolve rapidly with technology. Investments may also be embedded within broader health system, information and communications technology or development budgets, making them less visible than disease or programme-specific funding. These characteristics make dedicated classification and tracking systems especially important to capture the full scope of investments, assess gaps, coordinate stakeholders and ensure alignment with national priorities and health system goals. Doing so will help ensure the effectiveness and impact of digital health funding.

## Recommendations and pathways 

Here, we provide a set of recommendations to improve the tracking and accountability of digital health investments through the development and adoption of a digital health investment taxonomy.

### Developing a taxonomy

To address the challenges outlined in this article, a global digital health investment taxonomy could provide a common framework for categorizing and tracking investments across governments, development partners and private sector actors. The taxonomy could also be integrated into existing tracking and reporting mechanisms. The taxonomy would support coordination among funders, increase visibility of funding and gaps, enable evidence-based investment planning and create a foundation for accountability and learning.

Tracking financial inputs rather than outputs is important because the impacts of digital health investments are often indirect, long-term and shaped by multiple interventions, making outputs difficult to attribute to specific activities. Monitoring inputs provides a clearer basis for analysing investments. A standardized digital health taxonomy would enable these inputs to be systematically classified and tracked across stakeholders, with a particular focus on digital health planning and coordination.

The taxonomy would be developed through a review of existing frameworks and strategies, informed by expert input, and validated through stakeholder consultations and testing. The taxonomy could adopt a three-tier structure: high-level domains, defined investment areas and detailed subcategories, with cross-cutting tags (such as expenditure type, geography, equity) to support analysis.

### Adoption across stakeholders

For a digital health investment taxonomy to be effective, governments, development partners and private sector stakeholders should adopt it across the digital health financing ecosystem. Governments could use the taxonomy to plan, budget and track investments, while development partners and private sector actors could use it to align portfolios and support coordination. The taxonomy could also support country-level coordination and analysis of investment patterns.

### Integration in reporting mechanisms

A taxonomy could also support more comprehensive reporting on digital health within existing frameworks. The OECD Development Assistance Committee Creditor Reporting System could introduce dedicated purpose codes or policy markers (which indicate activities targeted to a policy objective) for digital health investments. At national level, incorporating digital health categories into the system of health accounts would enable governments to capture how funding on digital health moves through the system. Digital health should also be included as part of UHC monitoring at national and global levels to track digital health investments as an enabler of UHC progress, including in strengthening primary health care and equitable access to services. Together, these approaches would improve the visibility of digital health investments across both international and domestic data sets.

## Conclusion

Digital health is an important component of health systems, supporting integrated, efficient and equitable service delivery. Yet the absence of standardized definitions and tracking of digital health investments means that a comprehensive picture of the funding landscape is lacking. A digital health investment taxonomy could help address this gap by providing a shared framework for defining, categorizing and reporting digital health funding across stakeholders. Integrating such a taxonomy into national budgeting and financing, development partner and private sector investment planning, and international reporting systems could improve transparency, coordination and evidence-based decision-making.

By tracking investment in programme-specific interventions and cross-cutting capabilities, the taxonomy would improve visibility, coordination and accountability of investment across vertical initiatives and horizontal health system strengthening. In turn, these effects would support primary health care, resilient health systems and progress towards UHC.
